# LncRNA SNHG15 regulates autophagy and prevents cerebral ischaemia‐reperfusion injury through mediating miR‐153‐3p/ATG5 axis

**DOI:** 10.1111/jcmm.17956

**Published:** 2023-10-16

**Authors:** Yunhu Yu, Yunpeng Cai, Hang Zhou

**Affiliations:** ^1^ Neurosurgery Department People's Hospital of Honghuagang District of Zunyi Zunyi PR China

**Keywords:** ATG5, autophagy, cerebral ischaemia‐reperfusion injury, LncRNA SNHG15, miR‐153‐3p

## Abstract

Ischaemic stroke is a common cerebrovascular disease. Long non‐coding RNA (lncRNA) of small nucleolar RNA host gene (SNHG15) has been supposedly performed a regulatory role in many diseases. Nonetheless, the function of SNHG15 in cerebral ischaemia‐reperfusion injury has not been clarified. The OGD/R of Neuro2A cells simulated the ischaemic and reperfused states of the brain. Neuro2a cell line with stable transfection of plasmid with silent expression of SNHG15 was constructed. Neuro2a cell lines transfected with miR‐153‐3p mimic (miR‐153‐3p‐mimics) and miR‐153‐3p inhibitor (miR‐153‐3p‐inhibition) were constructed. Expression of SNHG15, mi R‐200a, FOXO3 and ATG7 in mouse brain tissue and N2a cells was identified by qRT‐PCR. Western blot (WB) analysis of mouse brain tissue and Neuro2a cells revealed the presence of the proteins ATG5, Cle‐caspase‐3, Bax, Bcl‐2, LC3 II/I and P62 (WB). The representation and distribution of LC3B were observed by immunofluorescence. The death of cells was measured using a technique called flow cytometry (FACS). SNHG15 was highly expressed in cerebral ischaemia‐reperfusion injury model. Down‐regulation of SNHG15 lead to lower apoptosis rate and decreased autophagy. Dual luciferase assay and co‐immunoprecipitation (CoIP) found lncRNA SNHG15/miR‐153‐3p/ATG5. Compared to cells transfected with NC suppression, cells transfected with miR‐153‐3p‐inhibition had substantially greater overexpression of LC 3 II/I, ATG5, cle‐Caspase‐3, and Bax, as determined by a recovery experiment, the apoptosis rate was elevated, yet both P62 and Bcl‐2 were significantly lower and LC3+ puncta per cells were significantly increased. Co‐transfection of miR‐153‐3p‐inhibition and sh‐SNHG15 could reverse these results. LncRNA SNHG15 regulated autophagy and prevented cerebral ischaemia‐reperfusion injury through mediating the miR‐153‐3p/ATG5 axis.

## 1 INTRODUCTION

Ischaemic stroke is a major disease that affects human health and threatens life.^1^ The disability caused by stroke leads to a severe strain on communities and families.^2^ The morbidity of cerebral stroke is 1114/100,000 in China.^3^ Almost 1.1 million Chinese people die of cerebral stroke every year and account for one‐third of global mortality.^4^ Cerebral stroke has ranked the ‘first killer’ of human life in China beyond coronary heart disease and tumour.^5^ Thus, improving prognosis of cerebral stroke is extremely urgent to increase prevention and treatment level of cerebral stroke and decrease mortality and disability rates. So far, revascularization has been the most effective method to treat acute ischaemic stroke.^6^ Because of the limited revascularization gap and subsequent ischaemia‐reperfusion damage in the ischaemic penumbra, the effectiveness of revascularization is unsatisfactory.^7^ Undoubtedly, exploration of the molecular mechanism and effective therapeutic targets of cerebral ischaemia reperfusion may overcome the limitations of current therapies and side effects.

Long non‐coding RNA (lncRNA) is a RNA that does not encode protein and contains >200 nucleotides.^8^ LncRNA does not encode protein, yet is involved in many important regulations, including chromosome reconstruction, genome‐wide imprinting, gene expression regulation, post‐transcriptional disruption and intranuclear transport of nucleic acids, at epigenetic, transcriptional and post‐transcriptional levels.^9^ A large amount of lncRNAs were found in central nervous system^10^ and played important roles in the development of brain. There is a lot of evidence show that ischaemic stroke is only one of many disorders of the neurological system that have been linked to lncRNA. For example, the continuous high level of expression of lncRNA SNHG15 indicates its ability to observe the dynamics of ischaemic stroke.^11^ LncRNA MEG3 mediates neuronal apoptosis through targeting miR‐424‐5p by MAPK signal pathway in ischaemic stroke.^12^ These evidences indicate great potential of lncRNA as a therapeutic target in ischaemic stroke. SNHG15 is located in chromosome 7p13 and found to accelerate cell proliferation and migration in Parkinson's disease^13^ and myocardial ischaemia reperfusion.^14^ SNHG15 has been studied in many diseases, but there is little research on cerebral ischemia‐reperfusion injury.

Autophagy is a cellular process that promotes lysosomal degradation of long‐life cytoplasmic proteins, and is activated in differentiation, nutrition deficiency or cell stress (including oxidative stress, endoplasmic reticulum stress and aggregation of protein aggregates).^15^ More and more evidence shows that autophagy plays an important role in a variety of cardiovascular diseases, such as aneurysm, aortic dissection, atherosclerosis, myocardial ischaemia‐reperfusion injury, etc. For example, knocking out ATG5 or ATG7, inhibiting autophagy and excessive activation of autophagy can promote the occurrence of aortic aneurysm and aortic dissection.^16^ Using computational modelling, the current investigation identified a possible regulatory effect for the lncRNA SNHG15/miR‐153‐3p/ATG5 pathway in ischaemic. For validating our speculation, we conducted this study.

## 2 METHODS AND MATERIALS

### 2.1 Cell culture

The mouse neuroblastoma cell line, Neuro2A, was obtained from the Chinese Academy of Medical Sciences and the Shanghai Institute for Life Science Cell Resource Center. Cultured cells had been kept up in Dulbecco's Modified Eagle's Medium (DMEM) supplemented with 10% fetal bovine serum (FBS, Gibco™, Catalogue No.: 16000044) in a 37°C incubator with 95% atmosphere and 5% CO_2_. When oxygen glucose deprivation/reoxygenation (OGD/R) cells were fused to 80%, pre‐treated DMEM (glucose, serum and oxygen free) was added rapidly and incubated for 6 h. Then cells were removed from the anaerobic box, the medium was discarded and addition of high‐glucose DMEM augmented by 10% fetal bovine serum. For the remaining investigations, the cells were kept in a 37°C incubator with 95% moisture and 5% CO_2_.

### 2.2 Cell transfection

A short hairpin interfering RNA (sh‐SNHG15) for SNHG15 and negative control (sh‐NC) were conceptualized and produced by GenePharma. The mimic of miR‐153‐3p (miR‐153‐3p‐mimics), the inhibitor of miR‐153‐3p (miR‐153‐3p‐inhibition) and respective negative controls (NC‐mimics, NC‐inhibition) were constructed. Transfection of these constructs into stable Neuro2A cells were performed with Lipofectamine 3000 kit (Invitrogen™) and culture medium containing G418.

### 2.3 Quantitative real‐time PCR (qRT‐PCR)

Extractions of RNA were performed after cells were collected utilizing the GenElute Total RNA Purification Kit (Sigma‐Aldrich; Merck KGaA). Reverse transcription of total RNA (2.0 μg) was performed with SuperScrip®Vilo cDNA synthesis kit (Invitrogen). The comparative overexpression of SNHG15, miR‐153‐3p, and ATG5 were determined employing qPCR with a SYBR green I Master Mix kit (Invitrogen; Thermo Fisher Scientific, Inc.) in a 7500 real‐time PCR system (Applied Biosystems; Thermo Fisher Scientific, Inc.). The methods followed what was laid forth in the kit's manual. U6 was the intrinsic control of miR‐153‐3p. GAPDH was the intrinsic control of SNHG15 and ATG5. The following cycling procedure was used for qRT‐PCR: initial denaturation, 95°C, 10 min; 95°C, 20 s, 60°C, 15 s and 72°C, 20 s; 40 cycles. Expression was quantified using the formula 2 −ΔΔCq. Primer sequences: SNHG15:Forward primer: 5′‐CAACCATAGCGGTGCAACTGTGC‐3′, Reverse primer: 3′‐GGCTGAACCAAGTTGCAAGTCATG‐5; miR‐153‐3p:Forward primer: 5‐GTCAATTGAGCACGTGGC‐ CAC‐3; Reverse primer:5‐GACGTACGGACTGACGGACCAC3; ATG5:Forward primer: CACCGAGTGGATAATCTTTATGGCA Reverse primer:AAACTGCCATAAAGATTATCCACTC; U6:Forward primer:GGAAGTAGCACCTGATTAGC Reverse primer:TTGGAATACGAATIGGCCG; GAPDH:Forward primer:CTCACCGGATGCACCAATGTT Reverse primer:CGCGTTGCTCACAATGTTCAT.

### 2.4 Western blot (WB)

Proteins from cells were extracted and run through an electrophoretic separation of sodium dodecyl sulfate polyacrylamide gel electrophoresis (SDS‐PAGE). After purifying the proteins, they were wiped onto a PVDF membrane, soaked in milk samples 5% at 37°C for 2 h, and finally at 4°C overnight. Antibodies included ATG5 antibody (1:1000), Cle‐caspase‐3 antibody (1:1000), BCL2 associated X (Bax) antibody (1:100), Bcl‐2 antibody (1:1000), Pro‐caspase‐3 antibody (1:1000), LC3 II/I antibody (1:1000), P62 antibody (1:1000) and GAPDH antibody (1:5000). Immunoreactive bands were detected by chemoluminescence (ECL reagent, Millipore) and X‐ray films were analysed for density. KD of specific protein: LC3 I 16kd, LC3 II 14kd, P62 48kd, ATG5 55kd, GAPDH 36kd, Cleaved Caspase‐3 17/19kd, pro Caspase‐3 35kd, bax 21kd, bcl2 28kd.

### 2.5 Cell apoptosis assay

Flow cytometry (FACS) using the Annexin V‐FITC/propidium iodide (PI) kit was used to analyse cell apoptosis (Sigma‐Aldrich, Merck KGaA). A 12‐well plate was seeded with transfected cells (3 × 10^4^ cells/well) and incubated at 37°C, 5% CO_2_ for 48 h. Subsequently, for 10 min in the darkness and at room temperature, the cells were stained with Annexin V‐FITC and PI. Cell apoptosis was analysed with the FACSCalibur flow cytometer (BD Biosciences) and FlowJo software (version 10, BD Biosciences).

### 2.6 Dual luciferase assay

Both wild type/mutant SNHG15 (SNHG15‐WT/MUT) and wild type/mutant Bax (ATG5‐WT/MUT) were established with DNA oligonucleotides and pMiR‐Reporter Vector. HEK293 cells were founder with miR‐153‐3p‐mimics and an untreated control to observe the effects of these adjustments (miR‐NC). Cells were cultured for 24 h before being harvested and tested for luciferase activation using a commercially available double luciferase assay kit (Promega).

### 2.7 RNA immunoprecipitation

RNA immunoprecipitation (RIP) was utilized by a EZ‐Magna RIP kit (Millipore, USA) as described in the instructions. Cells were collected and subject to lysis by RIPA. Using RIP wash buffer, beads coupled with anti‐AgO2 antibody (Millipool) or mouse IgG control were treated with total cellular protein isolate. Purified protease K was used to break down the protein found in the specimen. Immunoprecipitated RNA was extracted. In order to confirm presence of binding target, qRT‐PCR was used to examine isolated RNA.

### 2.8 Immunofluorescence detection

Treated Neuro2A cells were collected. PBS 1 mL was added into the culture dish for washing. Pre‐cooled methanol was added for fixation at −20°C for 15 min; Methanol was discarded, PBS wash, goat serum 5% was added for blocking at room temperature for 1 h; Blocking solution was discarded, first antibody (LC3B1, 1:100) was added for incubation at 4°C overnight; First antibody was discarded, subsequent to a wash with PBS, a secondary antibody (1:100) was inserted, and the substance was left alone for 2 h at room temperature and without light. After PBS wash, cells were incubated with DAPI at room temperature for 5 min; After PBS wash, antifluorescence quenching solution was added. Confocal microscopy pictures of cells were taken for analysis.

### 2.9 Statistical analysis

GraphPad 7 was used for all diagramming and statistics. SPSS 20.0 was used for the analysis of independent prognostic variables. To evaluate differences across groups, we used the *t*‐test for independent samples, while the paired *t*‐test was utilized to do in‐group differences for the mean ± SD of the measurements. Multi‐group comparisons were made using one‐way analysis of variance. The LSD *t*‐test was used for the inferential analysis after the fact. Where *p* < 0.05, statistical significance was present. *p < 0.05, **p < 0.01, ***p < 0.001,

## 3 RESULTS

**FIGURE 1:**
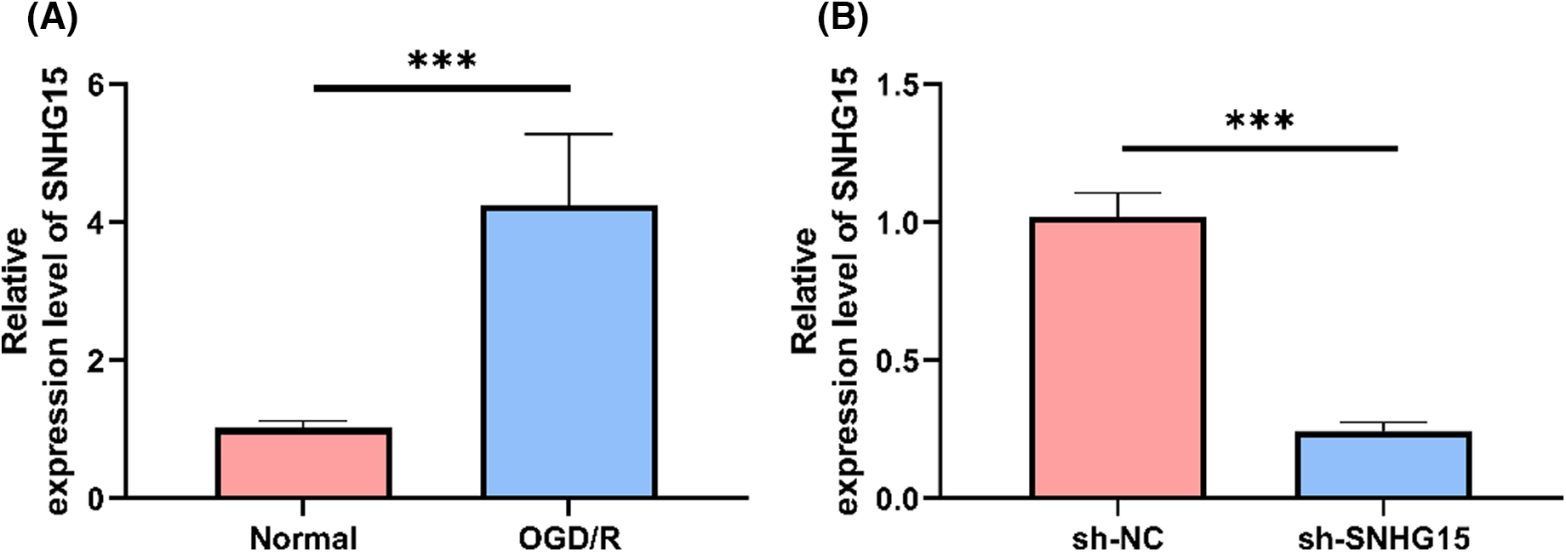
Intensity of expression of SNHG15 in OGD/R cells. (A) Relative expression level of SNHG15 in OGD/R cells by qRT‐PCR. (B) Relative expression level of SNHG15 in OGD/R cell model after transfection of sh‐SNHG15 by qRT‐PCR. **p < 0.01.

Expression of lncRNA SNHG15 in OGD/R cells. Examining SNHG15 expression in ischaemic stroke, we established a model of in vitro ischaemia‐reperfusion injury in Neuro2A cells by OGD/R. The expression level of SNHG15 in Neuro2A cells were significantly elevated, indicating that SNHG15 was regulated in ischaemia‐reperfusion injury, *p* < 0.001 (Figure 1A). Then we transfected sh‐SNHG15 in model cells. In comparison to sh‐NC cells, SNHG15 expression was dramatically reduced in sh‐SNHG15 cells, *p* < 0.001 (Figure 1B), indicating successful transfection of sh‐SNHG15 into OGD/R cells.

### 3.1 Down‐regulation of SNHG15 inhibits autophagy

**FIGURE 2:**
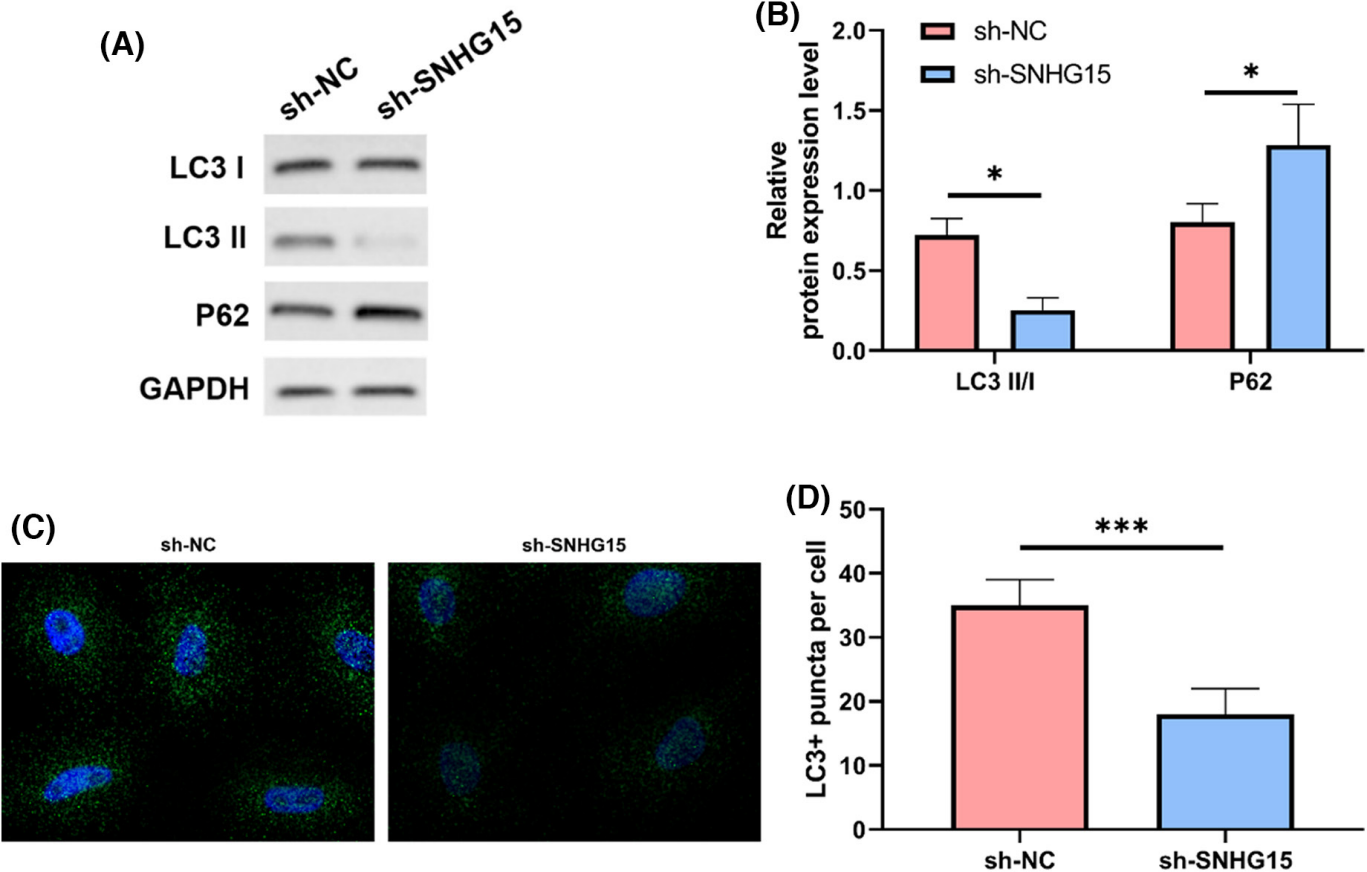
Inhibition on autophagy by sh‐SNHG15. (A) Protein bands. (B) P62 and LC 3 II/I detected by WB. (C) LC3 in cells identified by immunofluorescence. (D) Number of LC3+ cells highlighted by immunofluorescence. *p < 0.05, ***p < 0.001.

In order to observe the effect of regulating SNHG15 on autophagy, we detected the changes of P62 and LC 3 II/I proteins in cells transfected with sh‐SNHG15 and LC3+ puncta per cell by WB and immunofluorescence staining. Figure 2A,B shows that when compared with cells transfected with sh‐NC (*p* < 0.05), cells transfected with sh‐SNHG15 had considerably reduced LC3 II/I expression and substantially enhanced P62 expression (*p* < 0.05). Cells transfected with sh‐SNHG15 had considerably fewer LC3+ puncta per cell in contrast to sh‐NC‐transfected cells, *p* < 0.05, Figure 2C,D, as determined by immunofluorescence. These indicated that down‐regulation of SNHG15 inhibited autophagy.

### 3.2 Down‐regulation of SNHG15 inhibits apoptosis

**FIGURE 3:**
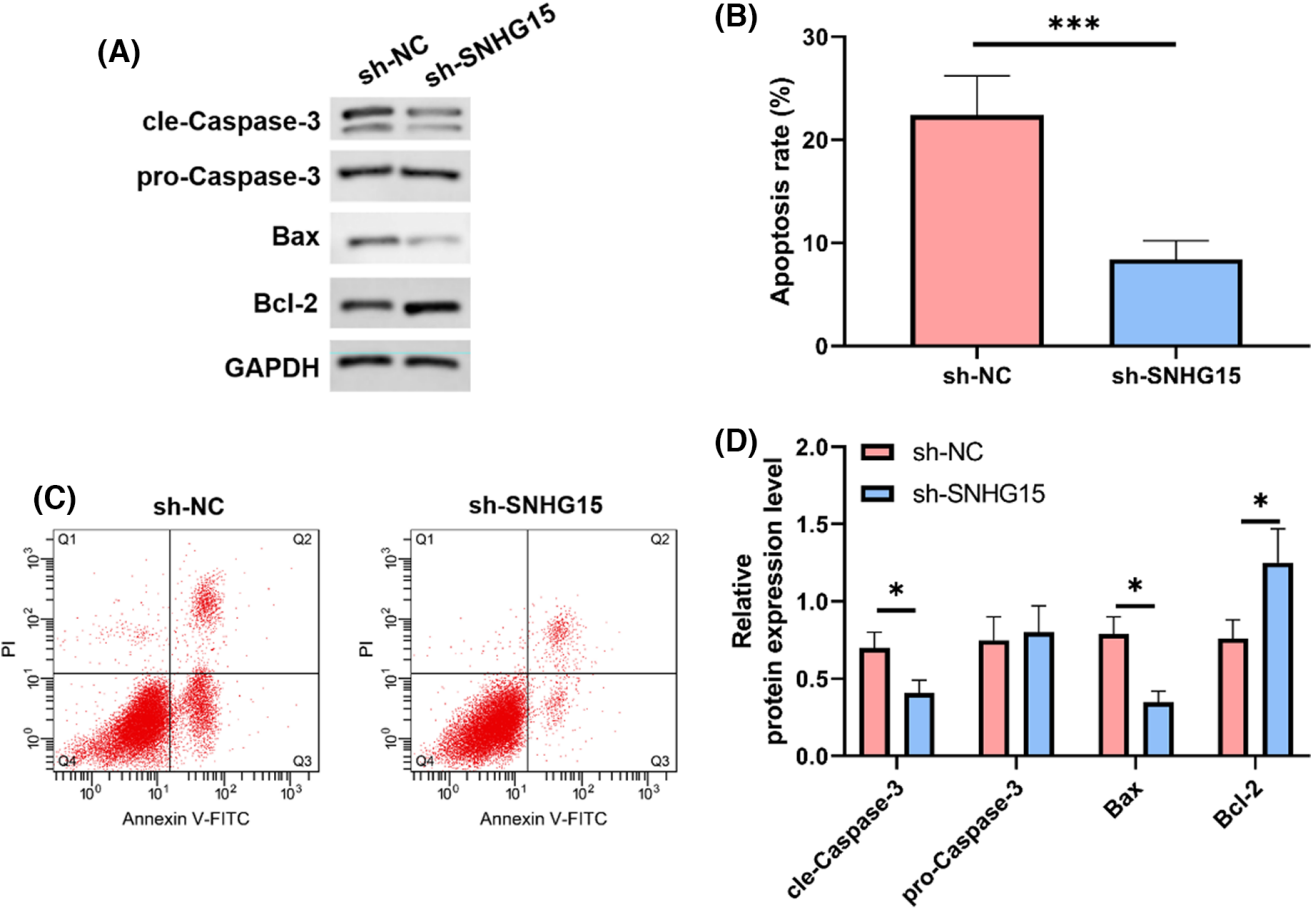
Inhibition of apoptosis by sh‐SNHG15. (A) FACS counting. (B) Apoptosis detected by FACS counting. (C) Protein bands. (D) Changes of cle‐Caspase‐3, Bax and Bcl‐2 detected by WB. *p < 0.05, ***p < 0.001.

Apoptosis is recognized as an important programmed cell death. In the present study, apoptosis of cells with transfection of sh‐SNHG15 was evaluated by FACS counting and WB. Figure 3A,B illustrates that flow cytometry demonstrated that cells implanted with sh‐SNHG15 had a significantly decreased apoptotic rate compared to cells transfected with sh‐NC (*p* < 0.05). WB demonstrated that the expression levels of both cle‐Caspase‐3 and Bax in cells with transfection of sh‐SNHG15 were significantly lower than those with transfection of sh‐NC, yet the expression level of Bcl‐2 was significantly elevated, *p* < 0.05, Figure 3C,D. This indicated that down‐regulation of SNHG15 inhibited apoptosis through regulating autophagy, and further prevented cells from ischaemia‐reperfusion injury.

### 3.3 SNHG15 was the sponge of miR‐153‐3p

**FIGURE 4:**
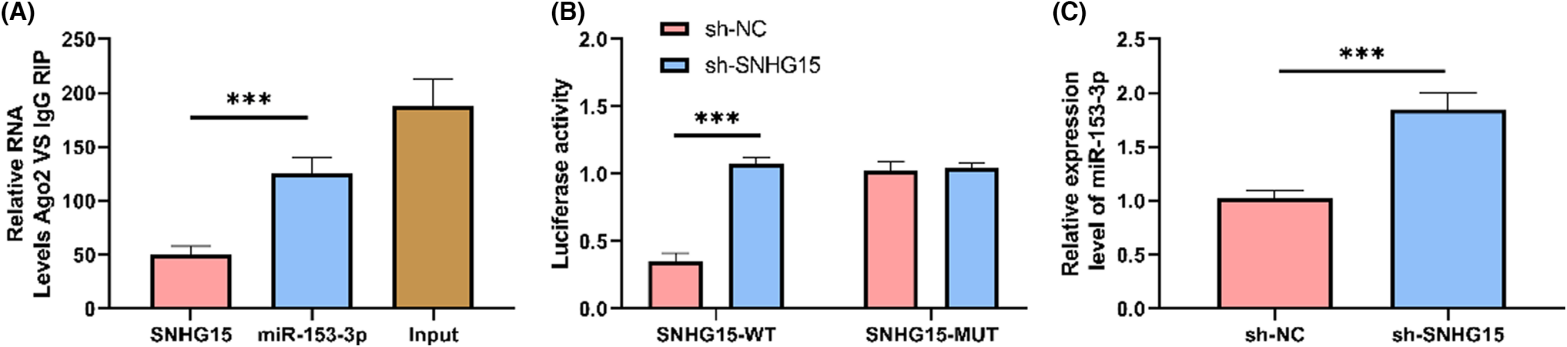
SNHG15 regulated miR‐153‐3p. (A) Immunocoprecipitation of SNHG15 and miR‐153‐3p by Ago2. (B) Dual luciferase assay detected the relationship between SNHG15 and miR‐153‐3p. (C) Using quantitative real‐time polymerase chain reaction, we determined the relative production of miR‐153‐3p in sh‐SNHG15 cells transfected. ***p < 0.001.

Estimated possible miR targets for SNHG15‐related mechanism investigation. Prediction by Starbase 3.0 showed targeted binding site in miR‐153‐3p for SNHG15. We validated the correlation between miR‐153‐3p and SNHG15 through immunocoprecipitation (CoIP) and dual luciferase assay. CoIP indicated that both miR‐153‐3p and SNHG15 were precipitated by Ago2 antibody rather than IgG, *p* < 0.05 (Figure 4A). Figure 4B demonstrates that in an SNHG15‐WT dual luciferase assay, miR‐153‐3p‐mimics dramatically suppressed luciferase output (*p* < 0.05). Moreover, relative miR‐153‐3p expression was considerably increased in cells transfected with sh‐SNHG15 (*p* < 0.05; Figure 4C,D) as determined by polymerase chain reaction quantification in real time. Together, according to these findings, SNHG15 could regulate miR‐153‐3p.

### 3.4 ATG5 was the target gene of miR‐153‐3p

**FIGURE 5:**
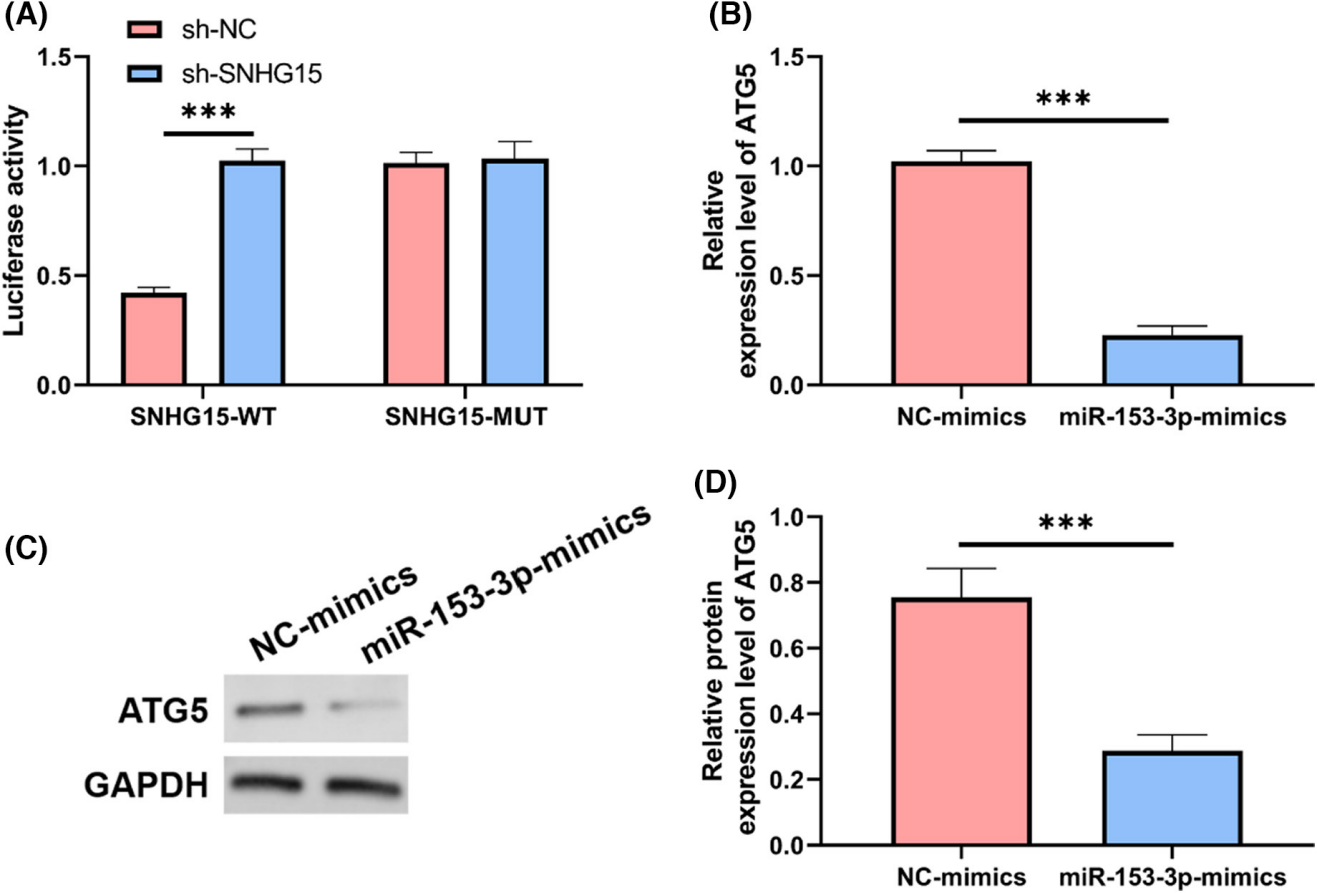
Targeted control of ATG5 by miR‐153‐3p. (A) The miR‐153‐3p and ATG5 targeting interaction was determined via a dual luciferase experiment. (B) Relative ATG5 expression was measured via miR‐153‐3p mimic transfection and direct answer polymerase chain reaction. (C). WB. (D) The proportional amount of ATG5 protein produced by miR‐153‐3p mimic‐transfected cells. ***p < 0.001.

MiR primarily modulates the transcription of downstream target genes. We found targeted binding sites in miR‐153‐3p for ATG5 through bioinformatic analysis. Based on dual luciferase assay and the expression of ATG5, we determined targeted binding between ATG5 and miR‐153‐3p, *p* < 0.05, Figure 5A. The mRNA and protein levels of ATG5 were also shown to be substantially reduced (*p* < 0.05) in cells transfected with miR‐153‐3p‐mimics, as determined by qRT‐PCR and WB (Figure 5B–D). These data suggested that miR‐153‐3p might control ATG5.

SNHG15 regulated autophagy and apoptosis through mediating miR‐153‐3p/ATG5 axis.

**FIGURE 6:**
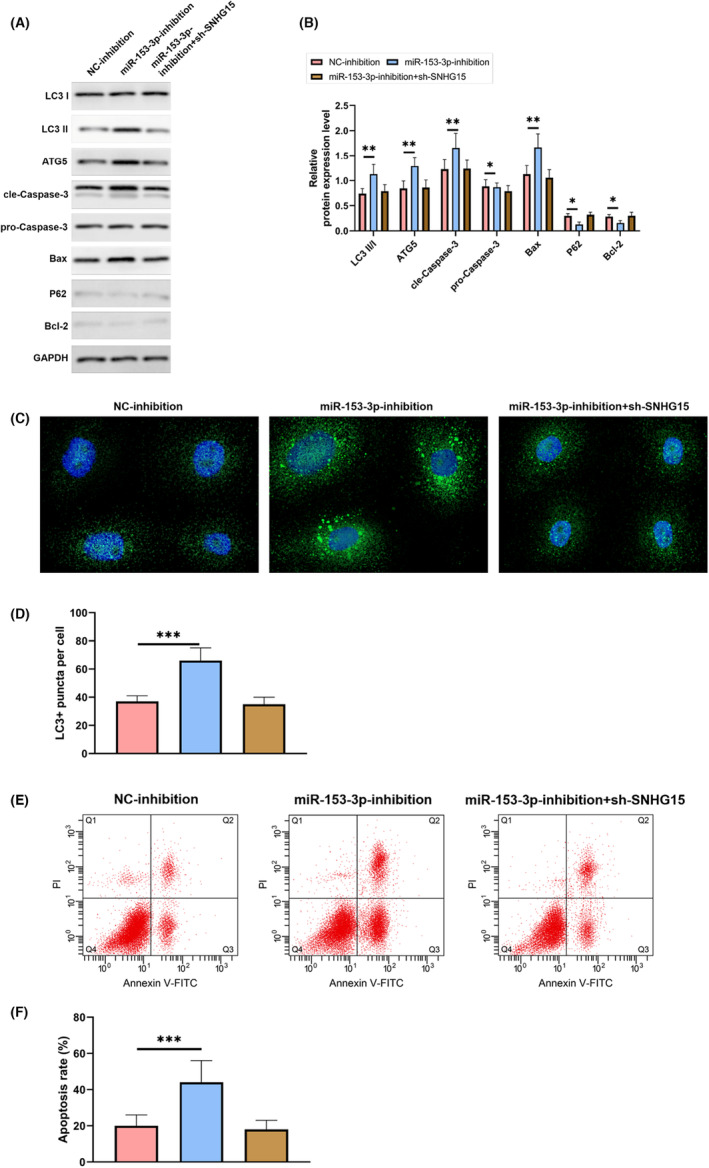
SNHG15 regulated autophagy and prevent apoptosis through mediating the miR‐153‐3p/ATG5 axis. (A) WB. (B) WB detected the change of protein level in cells after co‐transfection. (C) Immunofluorescence detected LC3 in cells. (D) Immunofluorescence detected LC3+ cells. (E) FACS counting. (F) FACS counting detected apoptosis in cells after co‐transfection. *p < 0.05, **p < 0.01, ***p < 0.001.

We utilized rescue assay to validate the mediation of miR‐153‐3p/ATG5 axis by SNHG15. Both WB and immunofluorescence showed that when compared to cells transfected with NC‐inhibition, miR‐153‐3p‐inhibition dramatically upregulated the transcription of LC 3 II/I, ATG5, cle‐Caspase‐3, and Bax, yet both P62 and Bcl‐2 were significantly lower and LC3+ puncta per cells were significantly increased, *p* < 0.05, Figure 6A–D. FACS counting showed that Figure 6E,F shows that as expected, cells transfected with miR‐153‐3p‐inhibition had a higher death rate than those infected with NC‐inhibition (*p* < 0.05). However, co‐transfection of miR‐153‐3p‐inhibition and sh‐SNHG15 could reverse these results. This indicated that SNHG15 could regulate autophagy and prevent apoptosis through mediating the miR‐153‐3p/ATG5 axis.

## 4 DISCUSSION

The mechanism of ischaemia‐reperfusion injury in acute ischaemic stroke is complex. The efficacy is not satisfactory even with revascularization. The residual disabling symptoms affect quality of life and even threaten human life.^17^ Autophagy is a process that the organelles and proteins that autophagosomes, which contain the material that has to be broken down, combine with lysosomes to produce autolysosomes, whereupon the contents are decomposed, so that cellular metabolism and renewal of some organelles are achieved to maintain intracellular homeostasis.^18^, ^19^

As a non‐encoding RNA, lncRNA has a length of >200 nucleotides. Due to limitation of scientific research level, lncRNA has been considered as a useless product of transcriptional metabolism for a long time. However, lncRNA is found to be important in epigenetics, transcription and post‐transcriptional regulation of gene expression.^20^ It was reported that lncRNA was closely associated with many diseases.^21^ The present study constructed an in vitro ischaemia‐reperfusion injury model of Neuro2A cells by OGD/R, and discovered that SNHG15 production was quite high. This indicated that an important function for SNHG15 in controlling the initiation and development of ischaemic stroke was demonstrated. Meanwhile, we found that down‐regulation of SNHG15 inhibited apoptosis and autophagy significantly in OGD/R model. This indicated that SNHG15 probably improve apoptosis through regulating autophagy. A previous study by Shen showed that knock‐out of SNHG15 could induce injury of N‐2a cells and inhibited apoptosis through inhibiting OGD, this was consistent with the present study.^22^ The present study further detected autophagy and found that knock‐out of SNHG15 may improve apoptosis through inhibiting autophagy.

The mechanism of SNHG15 in pancreatic cancer is unclear. An important mechanism is that lncRNA competes for miR element as a ceRNA and regulates genes.^23^ Probable binding sites in miR‐153‐3p and SNHG15 were determined in the current investigation using bioinformatics. To confirm the connection between miR‐153‐3p and SNHG15, we used a dual luciferase assay and RIP. Using a double luciferase experiment, we found evidence of potential targeted interaction between miR‐153‐3p and SNHG15. SNHG15 precipitated by Ago2 antibody was significantly higher than that by IgG. This indicated that SNHG15 may regulate miR‐153‐3p as a ceRNA. As an important mechanism, miR influences change of biological function through regulating mRNA. Specific interaction sites were identified in miR‐153‐3p and SNHG15 using bioinformatics techniques, and the association between miR‐153‐3p and ATG5 was confirmed using a dual luciferase test. After transfection of miR‐153‐3p‐mimics into cells, the expression levels of both mRNA and protein of ATG5 were significantly lower. As a result, miR‐153‐3p seemed capable of selectively regulating ATG5 expression.

We performed a rescue assay to validate that SNHG15 mediated miR‐153‐3p/ATG5 axis to regulate autophagy and apoptosis. Transfected cells with miR‐153‐3p‐inhibition had substantially increased degree of expressiveness of LC 3 II/I, ATG5, cle‐Caspase‐3, and Bax compared to NC‐inhibited cells, the apoptosis rate was elevated, yet both P62 and Bcl‐2 were significantly lower and LC3+ puncta per cells were significantly increased, *p* < 0.05, Figure 6A–D. Co‐transfection of miR‐153‐3p‐inhibition and sh‐SNHG15 could reverse these results. This indicated that SNHG15 could regulate autophagy and prevent apoptosis through mediating the miR‐153‐3p/ATG5 axis.

In their study, Liu et al. identified, in lncRNA SNHG15, a role similar to that of an absorbent for miR‐141, allowing osteosarcoma cells to proliferate, invade, and undergo autophagy.^24^ According to the literature, lncRNA SNHG15 has a role in autophagy‐mediated adriamycin resistance in osteosarcoma cells through addressing miR‐381‐3p/GFRA1.^25^ These studies demonstrated that SNHG15 regulated miR‐381‐3p/GFRA1 axis through autophagy. This research showed that via controlling autophagy, the SNHG15/miR‐153‐3p/ATG5 axis protected brain cells from damage caused by ischaemia and reperfusion. SNHG15/miR‐153‐3p/ATG5 axis was possible pathway and target in treatment of ischaemic stroke.

However, the present study had some limitations. First, we could not find any evidence of SNHG15/miR‐153‐3p/ATG5 gene or protein production in those who had had an ischaemic stroke. Whether expression of these genes was consistent with that in vitro model needs further experimental analysis. Second, the present study did not establish an in vivo model. Whether the same regulatory mechanism exists in animal models is unclear.

In conclusion, lncRNA SNHG15 could regulate autophagy and prevent cerebral ischaemia‐reperfusion injury through mediating the miR‐153‐3p/ATG5 axis.

## AUTHOR CONTRIBUTIONS

**Yunhu Yu:** Resources (equal); software (equal); supervision (equal); writing – original draft (supporting); writing – review and editing (equal). **Yunpeng Cai:** Data curation (equal); formal analysis (equal); writing – original draft (equal). **Hang Zhou:** Data curation (equal); formal analysis (equal); writing – original draft (equal).

## FUNDING INFORMATION

This study is supported by the National Natural Science Foun‐ dation of China (No. 81860450); Basic project in Huizhou Province (No. [2018] 1425).

## CONFLICT OF INTEREST STATEMENT

The authors declare that there are no conflicts of interest.

## Data Availability

The authors will supply the relevant data in response to reasonable requests.
